# Polyester-Based, Biodegradable Core-Multishell Nanocarriers for the Transport of Hydrophobic Drugs

**DOI:** 10.3390/polym8050192

**Published:** 2016-05-14

**Authors:** Karolina A. Walker, Jean-François Stumbé, Rainer Haag

**Affiliations:** 1 Institute of Chemistry and Biochemistry, Freie Universität Berlin, Takustr. 3, 14195 Berlin, Germany; karolina.walker@fu-berlin.de; 2 Laboratoire de Photochimie et d’Ingénierie Macromoléculaires, Université de Haute Alsace, 3 rue Alfred Werner, 68093 Mulhouse Cedex, France; jean-francois.stumbe@uha.fr

**Keywords:** nanocarrier, biodegradable, polyester

## Abstract

A water-soluble, core-multishell (CMS) nanocarrier based on a new hyperbranched polyester core building block was synthesized and characterized towards drug transport and degradation of the nanocarrier. The hydrophobic drug dexamethasone was encapsulated and the enzyme-mediated biodegradability was investigated by NMR spectroscopy. The new CMS nanocarrier can transport one molecule of dexamethasone and degrades within five days at a skin temperature of 32 °C to biocompatible fragments.

## 1. Introduction

**Figure 1:**
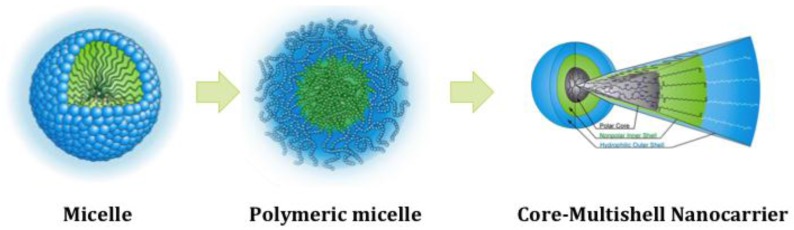
Evolution of nanocarriers, which has led to core-multishell nanocarriers. Adapted from PhD thesis M.Radowski and reprinted with permission from E. Fleige, B. Ziem, M. Grabolle, R. Haag, U. Resch-Genger, *Macromolecules* **2012**, 45, 9452–9459 [8]. © Copyright 2012 American Chemical Society.

A main objective for polymeric nanocarriers is to increase the solubility of hydrophobic and poorly water-soluble drugs. The idea to use nanocarriers originates from the mimicry of micellar architectures, which are self-assembled constructs made of amphiphiles. Inspired by micelles, the first attempts to mimic this architecture with polymeric structures was made in the late 1980s [1]. Apart from their good performance in terms of loading efficacy, both architectures can disassemble below a critical micellar concentration [2,3]. This drawback can be resolved by creating a micelle, as visualized in Figure 1, which is covalently linked at its focal point [4]. Although there have been advances in the field of unimolecular micelles, which are based upon hyperbranched and dendritic core molecules, such as hyperbranched poly(ethylene imine), hyperbranched polyglycerol, or hyperbranched polyester Boltorn^™^, little progress has been made in biodegradable systems [5,6]. The majority of published work focuses on solubilizing drugs in water-soluble nanocarriers. This feature of water solubility is introduced to the branched nanocarrier scaffold mostly by covalently attaching biocompatible poly(ethylene glycol) (PEG), which serves as the outer shell of the nanocarrier [7].

**Figure 2:**
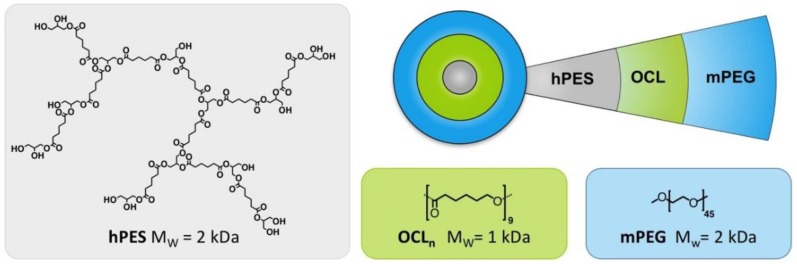
Building blocks and schematic representation of water-soluble CMS nanocarriers based on polyesters.

Our motivation was to create a biodegradable nanocarrier for the encapsulation of hydrophobic drugs, such as dexamethasone. As previously reported poly(ε-caprolactone) is a well-suited material for the entrapment or encapsulation of dexamethasone, we chose this linear, potentially biodegradable polymer to be a building block for the work presented here, as shown in Figure 2 [9,10]. Combining our expertise in the synthesis of hyperbranched polymers and synthesizing universal nanocarriers for the transport of drugs, our aim was to synthesize a biodegradable polyester-based core-multishell nanocarrier (CMS) for the encapsulation of dexamethasone [5,6,11]. In this paper we will show the synthesis of a CMS based on a hyperbranched polyester (shown in Figure 2) that acts as a multifunctional initiator for the ring-opening polymerization of ε-caprolactone (ε-CL) in a grafting-from approach, which is terminated by a methoxy poly (ethylene glycol) (mPEG) chain to introduce the feature of water solubility. The synthesis comprises a surprising, and not often discussed, ring opening of glycidol by the terminal carboxylic acid groups of the core. This leads to the introduction of hydroxyl groups at the surface of the inner core and will be discussed based on detailed analysis. We studied how the polyester architecture can transport the drug dexamethasone and the degradation behavior of the CMS nanocarrier.

## 2. Materials and Methods

### 2.1. Materials

Adipic acid (Fluka Analytical, Steinheim, Germany), dibutyltin dilaurate (DBTL, Merck KGaA, Darmstadt, Germany), Tin(II) 2-ethylhexanoate (Sn(Oct)_2_, 92.5%–100%), glycidol (Sigma Aldrich, Steinheim, Germany), 1-(3-dimethylaminopropyl)-3-ethylcarbodiimide hydrochloride (EDC, ≥99%, Carl Roth GmbH + Co. KG, Karlsruhe, Germany), 4-(dimethylamino)-pyridin (4-DMAP, 99%, Acros Organics, Acros Organics, Beel, Belgium), poly (ethylene glycol) methyl ether (mPEG, *M*_n_ ~2000 g·mol^−1^_,_ Sigma Aldrich, Steinheim, Germany), and succinic anhydride (Acros Organics, Beel, Belgium) were used without further purification. ε-Caprolactone (ε-CL, 97%, Sigma Aldrich, Steinheim, Germany) was dried over ground CaH_2_ (Sigma Aldrich) and distilled using cryogenic distillation prior to reaction. Solvents were purchased as HPLC grade and used without further purification. Anhydrous solvents were either purchased as ultra-dry solvents from Acros Organics, or taken from a MBraun MB SPS-800 solvent purification system. CAL B lipase, which is immobilzed on acrylic resin, was purchased from Sigma Aldrich. Dialysis was performed in benzoylated cellulose dialysis tubes from Sigma-Aldrich (width: 32 mm, MWCO = 1000 g/mol). Ultrafiltration was performed in a Millipore solvent-resistant stirred cell (47 mm diameter, 300 mL volume) with Millipore-regenerated cellulose membranes (47 mm diameter) and nitrogen pressure set between four to five bar. Reactions were performed under dry conditions using a Schlenk line, Schlenk flasks, and argon as the inert gas.

### 2.2. Characterization

#### 2.2.1. Nuclear Magnetic Resonance Spectroscopy

^1^H NMR and ^13^C NMR spectra were recorded on a Bruker AMX 500 (Bruker Corporation, Billerica, MA, USA), Jeol ECP 500 (JEOL (Germany) GmbH, Freising, Germany), or a Bruker Avance 400 spectrometer (Bruker Corporation, Billerica, MA, USA) (at 295 K). Inverse-gated ^13^C NMR and overnight measurements spectra were recorded on a Bruker Avance 500 spectrometer, or a Bruker Avance III 700 (Bruker Corporation, Billerica, MA, USA). Tetramethylsilane was used for internal calibration at 125 MHz with complete proton decoupling. Degree of branching was evaluated by inverse-gated ^13^C NMR according to Frey *et al.*, as shown in Equation (A4) in the Appendix [12].

#### 2.2.2. TAV and THV

Total acid values TAV and total hydroxyl values THV were determined based on ^1^H NMR spectroscopy (see Appendix).

#### 2.2.3. Gel Permeation Chromatography

GPC measurements in THF were performed with highly-dilute fractions eluting from a SEC system consisting of an Agilent 1100 solvent delivery system (Agilent Technologies, Santa Clara, CA, USA) with isopump, manual injector, and an Agilent 1100 differential refractometer. Three 30 cm columns were used (PLgel Mixed C, Agilent Technologies, Santa Clara, CA, USA, 5 μm particle size) to separate polymer samples. The mobile phase was THF and the flow rate was 1.0 mL·min^−1^. The columns were held at room temperature. For each measurement, 100 μL of a sample of 15 or a 5 mg mL^−1^ solution was injected. WinGPC Unity from PSS was used to acquire data from the seven scattering angles (detectors) and the differential refractometer. Molecular weights and molecular-weight distributions were compared with poly(methyl methacrylate) (PMMA) standards (PSS, Mainz, Germany).

GPC in DMF data was obtained by measurements using a Shimadzu (Kyoto, Japan) liquid chromatography (LC) set up with degasser, pump, auto sampler, column oven, and differential refractometer. Three PolarSil columns (PSS Polymer Standards Service GmbH, Mainz, Germany; PolarSil 8 mm × 300 mm, 100, 1000, 3000 Å with 5 μm particle size) using DMF with 0.3% LiBr and 0.6% acetic acid as the mobile phase at a flow rate of 1 mL·min^−1^ were used to analyze polymer samples. The columns were operated at 40 °C with the RI detector set to the same temperature. The calibration was performed by using polystyrene standard (PSS, Mainz, Germany). Samples were measured at a concentration of 10 mg·mL^−1^ injecting 100 μL. LC solution software from Shimadzu was used for data acquirement and interpretation.

#### 2.2.4. Dynamic Light Scattering

DLS experiments were performed using a Malvern Zetasizer Nano instrument (Malvern Instruments Ltd, Worcestershire, UK) equipped with a He-Ne laser (633 nm) using backscattering mode (detector angle 173°). The CMS nanocarriers were dissolved in dH_2_O, mixed by a Vortex shaker for 2 min, followed by filtration using a 0.45 μm RC syringe filter. 100 μL of the filtered solution was added to a disposable Plastibrand® micro cuvette (Brand GmbH + Co KG, Wertheim, Germany) with a round aperture. The autocorrelation functions of the backscattered light fluctuation were analyzed using Zetasizer DLS software from Malvern Instruments Ltd (Worcestershire, UK) to determine the size distribution by intensity and volume. The measurements were performed at 25 °C, equilibrating the system on this temperature for 120 s. Mean diameter values were obtained from four different runs.

#### 2.2.5. High Pressure Liquid Chromatography

HPLC measurements for the analysis of dexamethasone content was performed on a Knauer Smartline-HPLC system with an internal UV absorption detector (λ = 254 nm), equipped with a Gemini RP C18 column (Phenomenix, 250 nm × 4.6 mm, particle size: 5 μm) and run with an acetonitrile-water (40:60) mixture as the mobile phase at a flow rate of 1.0 mL·min^−1^ under isocratic regime. The data were analyzed with Chromgate software (Knauer, Berlin, Germany). A calibration curve of dexamethasone was obtained by measuring dexamethasone in an acetonitrile-water (40:60) mixture in the concentration range of 0.004–2 mg·mL^−1^.

#### 2.2.6. Infrared Spectroscopy

IR-spectra were obtained from a Nicolet Avatar 320 FT-IR spectrometer (Thermo Fisher Scientific Inc., Waltham, MA, USA) operating from 4000–400 cm^−1^, equipped with a Smart Orbit ATR accessory with a diamond crystal. 

### 2.3. Synthesis

#### 2.3.1. Synthesis of Hyperbranched Polyester hPES **1**

**Figure A6:**
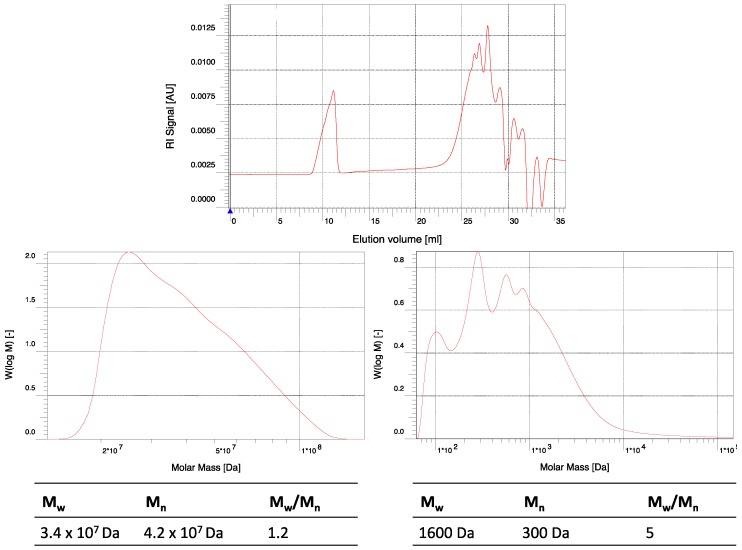
GPC elugram of crude hPES 1 in THF. (Top) full range elugram; *y*-axis: elution volume. (Bottom) detailed elugrams with molecular weights; *y*-axis: molar mass.

At room temperature, adipic acid (39.9 g, 273 mmol, 1.2 eq) was charged into a three-neck glass vertical reactor, equipped with a mechanical stirrer and a Liebig condenser. After adding pre-dried glycerol (20.9 g, 228 mmol), the bulk monomer mixture was heated to 150 °C. Under stirring at 150 rpm, a 0.6 mL of a stock solution of DBTL in toluene (100 ppm) was added to the molten monomers using a syringe. The reaction temperature was increased to 160 °C. After 1 h at 160 °C, the formed volatiles were removed by cryo distillation and collected in a round-bottom flask. The removal of volatiles was repeated every hour. With proceeding reaction time, the frequency of volatile removal was increased to once per every 10 min. Conversion of the reaction was controlled by determination of the ratio of unreacted to total amount of acid groups, using ^1^H NMR spectroscopy. When conversion almost reached the maximum conversion *P*_A_ as determined by the Flory Equation, the reaction was stopped by complete removal of the volatiles and cooling the reactor to room temperature. The viscous product hPES **1** was obtained without any further purification as a light yellow viscous solid and dissolved in THF for easier handling. Product was characterized via ^1^H NMR, IG ^13^C NMR, and GPC in THF (see Table A1 and Figure A6).

#### 2.3.2. Synthesis of Polyesterol hPES-OH **2**

**Figure A7:**
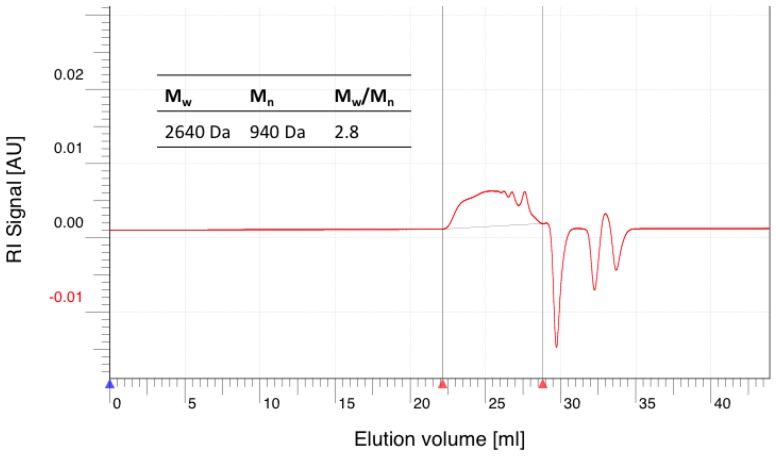
GPC elugram of purified hPES-OH 2 in THF.

30 mL of a solution of hyperbranched polyester hPES **1** in THF (c = 347 mg·mL^−1^, 10.41 g, 19 mmol COOH) was charged into a Schlenk flask. Residual catalyst DBTL (1.3 mg, 3.9 μmol) from the original product hPES **1** was used to catalyze the reaction; no further catalyst was added. After solubilization in 10 mL DMF under stirring at RT, the flask was heated to 85 °C. THF was removed from the mixture under controlled reduced pressure using cryo distillation. Within the limits of ^1^H–NMR, no residual THF was detected. After that, the mixture was heated to 110 °C. Glycidol (1.25 mL, 1.388 g, 19 mmol, 1 eq) was added dropwise to the stirring yellow solution during a time period of 10 min using a syringe. The reaction mixture was stirred at 110 °C for 120 min, afterwards at RT overnight. Due to the incompleteness of the reaction, the reaction was heated to 110 °C again and more glycidol (0.1 mL, 20 mmol in total) was added dropwise to the stirring reaction mixture. The reaction was stirred at 110 °C for 5.5 h and afterwards allowed to cool down to RT. The viscous product was obtained without further purification and stored in DMF. Product was characterized via ^1^H NMR, IG ^13^C NMR, and GPC in DMF (see Figure A7).

#### 2.3.3. Synthesis of Linear Di-Block Copolymer hPES-OCL_9_-OH **3**

**Figure A8:**
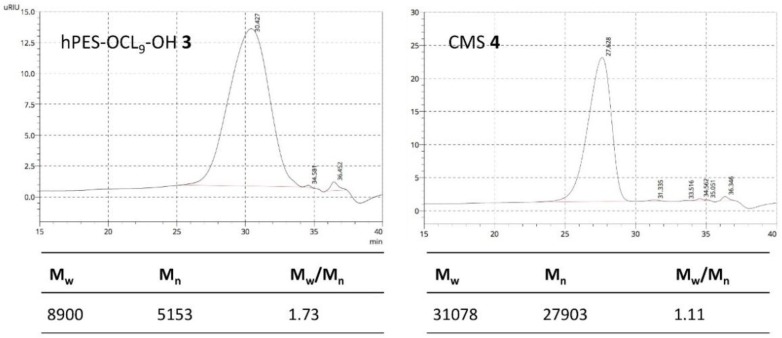
GPC elugram of purified hPES–OCL9–OH 3 and CMS 4 in DMF.

In a Schlenk flask pre-dried macroinitiator hPES-OH **2** (2 g, 10.6 mmol OH) was dissolved in freshly-distilled ε-caprolactone (6.18 g, 54 mmol) at 60 °C and two drops of Sn(Oct)_2_ were added to the stirring mixture, followed by an increase of the temperature to 125 °C. The bulk mixture was stirred at 125 °C for 18 h. Purification was performed by dissolving the crude reaction mixture in DCM in high dilution and precipitation in a high excess of ice-cold MeOH under vigorous stirring. The dispersion was separated from the formed gel, solvent was removed under reduced pressure, and the received solid was redissolved in DCM in high dilution. Another precipitation was performed by adding the DCM solution drop-wise into vigorously stirred ice-cold diethyl ether. The mixture was separated by centrifugation at 4000 min^−1^ for 1 min, and the supernatant was collected. After drying the separated supernatant under reduced pressure, the received solid was once again purified using dialysis in DCM (benzoylated RC membrane, MWCO = 1000 g/mol, 7 h) for removal of Sn(Oct)_2_ and traces of ε-caprolactone. A white wax-like solid product was obtained after removal of solvent under reduced pressure (2.69 g, yield: 33%). The product was characterized via ^1^H NMR, IG ^13^C NMR, and GPC in DMF (see Figure A8).

#### 2.3.4. Functionalization of mPEG-OH

To a solution of pre-dried mPEG (4.786 g, 2.5 mmol) in a mixture of 10% anhydrous DMF in anhydrous THF (28 mL) in a 50 mL Schlenk flask, 4-DMAP (0.44 g, 3.8 mmol, 1.5 eq), TEA (0.5 mL, 3.8 mmol, 1.5 eq), and succinic anhydride (1.2 g, 12.5 mmol, 5 eq) were added under stirring at RT. After stirring at RT for three days, unreacted precipitated starting material was removed from the solution and the solution was dried under reduced pressure. The crude product was purified by precipitation from DCM solution into ice-cold, 10-fold excess of Et_2_O. The formed precipitate was filtered off using a glass filter (P4), redissolved in DCM, and precipitated once more following the same procedure. The collected precipitate was dried under high vacuum and 3.5 g (1.67 mmol, yield: 70%) of pure product were obtained. The product was characterized via ^1^H NMR, IG ^13^C NMR, and GPC in DMF.

#### 2.3.5. Synthesis of Core Multishell Nanocarrier hPES-OCL_9_-PEG-OMe **4**

In a dried 25 mL Schlenk flask, solid mPEG-COOH (1.110 g, 0.529 mmol, 1.1 eq) was added at RT to a stirring solution of hPES-OCL_9_-OH **3** (304 mg, 0.48 mmol OH) in 6 mL anhydrous DMF. After the addition of 4-DMAP (0.016 g, 0.106 mmol, 20 mol %), EDCl (0.110 g, 0.574 mmol, 1.1 eq) was added at 0 °C. The reaction mixture was stirred for 10 min at 0 °C, and then allowed to reach RT by removing the ice bath. After 20 h of stirring at RT, the crude product was purified via extensive ultrafiltration (DMF, MWCO = 10,000 g/mol), followed by repeated fractionation. For this purpose, the impure product was dissolved in DCM and yielded a clear solution. Hexane was added to the clear solution at RT until cloudiness appeared. The cloudy dispersion was heated to obtain a clear solution, followed by the addition of hexane to obtain a dispersion. The warm solution was allowed to reach RT and centrifuged (1 min, 3900 min^−1^) to separate into a stable dispersion and sediment. The dispersion was dried and refractionated following the above-described procedure. The progress of purification was monitored using GPC in DMF. After six cycles of refractionation and removal of solvent under reduced pressure, followed by drying at high vacuum, a white solid product was obtained (0.116 g, yield: 12%). The product was characterized via ^1^H NMR, IG ^13^C NMR, and GPC in DMF (see Figure A8).

#### 2.3.6. Encapsulation of Dexamethasone in CMS Nanocarrier

Encapsulation of dexamethasone was performed using the film method uptake [8], in which dexamethasone solubilized in ethanol was dried under reduced pressure at 40 °C, which created a dry film at the bottom of the vial. The dry film was subsequently dissolved in a double amount of CMS nanocarrier in dH_2_O at a concentration of 10 mg·mL^−1^. The mixture was stirred for 22 h at 1200 min^−1^ and RT and, afterwards, filtered using a 0.45 μm PTFE syringe filter to remove excess dexamethasone. The size of the loaded CMS nanocarriers was determined using a DLS measurement of the aqueous solutions after filtration. The determination of dexamethasone content via HPLC was performed either by aqueous solution after filtration, to which acetonitrile (ACN/dH_2_0 4:6) was added prior to measurement, or the samples were first freeze-dried and then redissolved in an acetonitrile-water (4:6) mixture before measurement. The amount of dexamethasone in the CMS solution was determined by HPLC measurement relative to a dexamethasone calibration curve. As dexamethasone has some solubility in water, a blank sample was prepared to determine the natural solubility of dexamethasone in water. Therefore, a solution of dexamethasone in water was prepared in a procedure analog to the film method uptake. The solubility of the drug obtained from this blank control was subtracted from the nanocarrier results in order to obtain the effective loading. The values obtained from HPLC measurements of dexamethasone-loaded CMS were inserted in the following equation, which led to the loading capacity (LC):

(1) $$\text{Loading capacity}\;\left(LC\right)=\frac{n\left(\text{encapsulated dexamethasone}\right)}{n\left(\text{nanocarrier}\right)}\;\times 100$$

#### 2.3.7. Degradation of CMS Nanocarriers

Acidic degradation of CMS nanocarriers was studied by incubation of 2 mL of a CMS nanocarrier solution (10 mg·mL^−1^) in acetate buffer (pH 5.0) at 32 °C and constant stirring at 500 rpm. At defined time points, samples of 100 μL were withdrawn and freeze-dried in 1.5 mL Eppendorf tubes. The obtained lyophilisates were dissolved in DMSO-d_6_. Insoluble salts were separated by centrifugation at 4000 U·min^−1^ for 1 min, and the supernatant was analyzed by ^1^H NMR spectroscopy. For studying enzymatic degradation, samples were prepared for each time point as follows: 0.5 mL of a CMS nanocarrier solution (5 mg·mL^−1^) in 0.1 M PBS buffer (pH 7.4) was prepared in a 1.5 mL Eppendorf tube, and 200 wt % with respect to the polymer of immobilized CAL B was added, followed by 5 μL of n-butanol. The samples were incubated at 32 °C in an incubator shaker (New Brunswick Scientific Co. Int., Enfield, CT, USA). Samples were withdrawn at defined time points, frozen with liquid nitrogen, and kept in a freezer. After all the samples were obtained, the samples were allowed to thaw, and the immobilized enzymes were separated from the aqueous solution by centrifugation at 4000 U·min^−1^ for 1 min. The supernatant was transferred to a 1.5 mL Eppendorf tube, and combined with 0.5 mL dH_2_O used for washing the separated resin. The resin-free aqueous solution was freeze-dried, and the obtained lyophilisate was dissolved in DMSO-d_6_. After the insoluble salts were separated by centrifugation (4000 U·min^−1^, 1 min), the supernatant was analyzed via ^1^H NMR spectroscopy. As a control for both experiments, degradation of CMS nanocarriers in 0.1 M PBS buffer (pH 7.4) was performed in the absence of enzyme by incubation of 1 mL of a CMS nanocarrier solution (10 mg·mL^−1^) in 0.1 M PBS buffer (pH 7.4) at 32 °C and constant stirring at 500 rpm. At defined time points, samples of 100 μL were withdrawn and freeze-dried in 1.5 mL Eppendorf tubes. The obtained lyophilisates were dissolved in DMSO-d_6_. Insoluble salts were separated by centrifugation at 4000 U·min^−1^ for 1 min, and the supernatant was analyzed by ^1^H NMR spectroscopy.

## 3. Results and Discussion

### 3.1. Synthesis of Hyperbranched Polyester

**Scheme 1:**
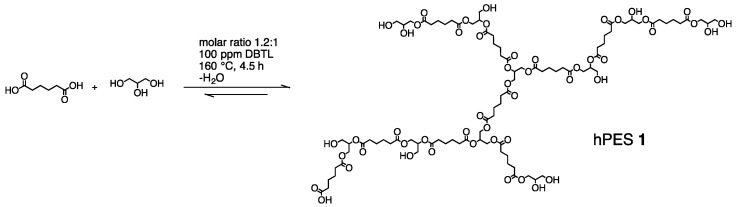
Synthesis of hyperbranched polyester hPES **1** used for the synthesis of CMS nanocarriers as a branching scaffold.

Based on previous work of Stumbé *et al*., hyperbranched polyester hPES **1** was synthesized, as shown in Scheme 1, by polycondensation of a 1.2:1 molar ratio of adipic acid and glycerol at 160 °C and catalyzation with dibutyltin dilaurate [13]. NMR spectroscopy, which is the key for evaluating the hyperbranched polymer’s structure, was used during the polymerization process to control the polymerization conversion, and to provide information about the extent of branching. The first step to control the polymerization via NMR was to predict the theoretical polymerization conversion *P*_A_ before reaching the gel point. This value could be calculated based on Flory’s theory of gelation and led to a *P*_A_ value of 0.79 for a molar ratio of 1.2:1 (diacid:triol) [14].

**Figure 3:**
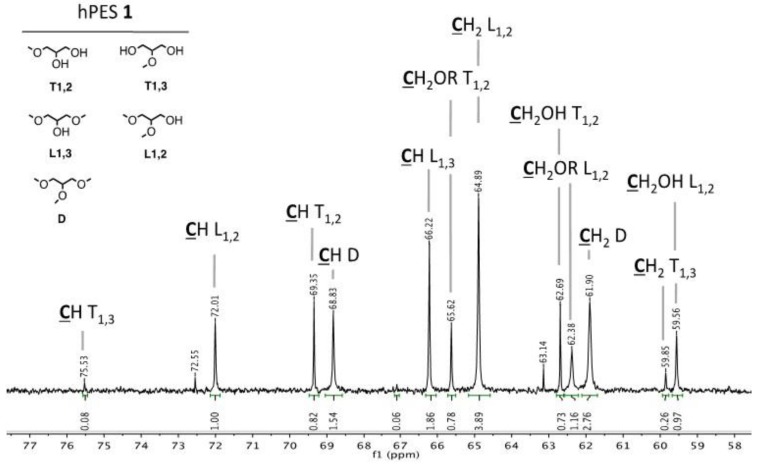
Extract of an IG ^13^C NMR spectrum of hPES-OH **2** that is crucial for evaluation of DB and shows the assignment of methine ***C***H and methylene ***C***H2 signals of various glycerol units.

The conversion P was monitored by ^1^H NMR spectroscopy based on the conversion of acid to ester groups, *i.e.*, the ratio between the integral of the methylene next to the acid at 2.2 ppm and the integral of the methylene next to the ester at 2.3 ppm. The calculated P value of 0.83 is slightly higher than the *P*_A_ value of 0.79. The reason might be the underestimation of the reactivity difference of primary and secondary alcohol functional groups of glycerol in Flory’s theory. Information about the degree of branching is extracted from inverse-gated ^13^C NMR (IG ^13^C NMR). The degree of branching (DB) of a hyperbranched polymer contributes the most to an understanding of the polymers’ architecture, because it reflects the perfection of the branching, which is essential for evaluating the structural similarity to dendritic systems. DB values range from 0 for linear systems to 1 for perfectly-branched dendrimers [15]. In hPES **1**, glycerol is the trifunctional branching unit (B_3_) and, thus, mainly responsible for the extent of branching. If the trifunctional branching unit has equal reactivities at every reactive end group, full branching is achieved at every step. In reality, the primary alcohol groups of glycerol are reported to have high reactivity, while the secondary alcohol shows reduced reactivity [16]. This difference in reactivity leads to the formation of different types of distinguishable glycerol units. Glycerol can react in three modes: at one primary and one secondary alcohol group forming a **L_1,2_** unit, at both primary alcohol groups forming a **L_1,3_** unit, and it can react with all three functional groups, which leads to a branching unit **D**. Terminal glycerol units can either have one free primary and one free secondary alcohol group that produce a **T_1,2_** unit or two free primary alcohol groups, which form **T_1,3_**, as shown in Figure 3. According to previously published detailed analysis, and as supported by HMQC-NMR spectroscopy, the signals of the five types of glycerol units obtained by IG ^13^C NMR spectroscopy can be assigned, leading to the DB value as defined by Frey *et al.* [12,13,17]. Based on the spectrum shown in Figure 3, a DB value of 0.52 was calculated for product hPES **1**. Values for total acid and total hydroxyl values were obtained by ^1^H NMR spectra analysis. The majority of free functional groups are represented by free hydroxyl groups with a percentage of 56%, compared to the overall amount of free functional groups. Molecular weights were determined by GPC measurements in THF and showed two peaks with values of 3.7 × 10^7^ and 1600 g·mol^−1^ in *M*_w_.

### 3.2. Modification of hPES **1** to Uniform Diol Endgroups

**Scheme 2:**
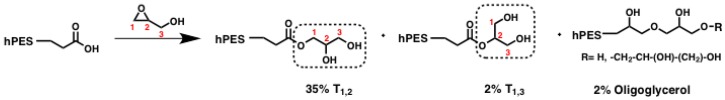
A simplified scheme of polyester modification by ring-opening reaction of glycidol; crucial units for NMR evaluation are highlighted in boxes; %: abundance of species relative to abundance of all glycidol units.

In order to obtain a monofunctional branched polyester scaffold, hPES **1** was modified with glycidol via ROP, which yielded hPES-OH **2**. The benefit of using glycidol is that the risk of transesterification during the process is omitted and the number of hydroxyl groups increases rapidly, as one ring-opening process yields two hydroxyl groups in the first ring opening step. The new approach in this synthesis is that glycidol reacts with carboxylic acids, instead of hydroxyl groups. Reactions between carboxylic acids and glycidol have not been reported in literature yet. Nevertheless, there are some reports of comparable reactions with epoxide moieties and carboxylic acids, which took place under metal-mediated catalysis. The reported catalysts for the ring-opening reactions of epoxides initiated by carboxylic acids are generally limited to Lewis acid catalysts, such as FeCl_3_, tetrabutylammonium chloride and bromide, and Ce(OTf)_4_ [18,19,20,21]. In the case of glycidol, the combination of the high ring strain of the epoxide and the polarity of the carbon-oxygen bonds makes the ring prone to nucleophilic attack. The ring-opening reactions can be catalyzed under acidic or basic conditions, but can also take place at high temperatures initiated by any weak nucleophile. The latter case is called thermally-induced, ring-opening reaction of glycidol [22]. Based on the above-mentioned literature, we hypothesized that DBTL as Lewis acid mediates a ring-opening of glycidol initiated by carboxylic acid of hyperbranched polyesters. Hyperbranched polyester hPES **1** with an total acid value of 1.8 mmol carboxylic acid groups per gram polyester was modified with equimolar amounts of glycidol per hydroxyl group of hPES **1**, aiming at a modification of one glycidol per carboxylic acid. We chose the polar aprotic solvent DMF as solvent, since preliminary results showed that it performed best for this type of ring-opening reaction. The reactions were performed at a bath temperature of 110 °C for two hours and afterwards at room temperature overnight. The reaction progress was monitored by ^1^H NMR, which showed the disappearance of the methylene signal next to the free carboxylic acid as well as the proton signal of terminating carboxylic acid groups. We paid special attention to the expected changes of several structural units as well as the possibility of the shown side reaction, as shown in Scheme 2.

^1^H and inverse-gated ^13^C NMR spectra were measured and checked for a change in methine and methylene signals of the various glycerol units. In summary, the amount of esterified carboxylic acid units increased by a factor of 2.5 compared to the esterified carboxylic acid units before the modification, while full modification of carboxylic acids could not be achieved. The degree of functionalization of carboxylic acids is *D*_f_ (COOH) = 0.83. The ring-opening of glycidol did not only take place at C1 carbon atom, which led to a new T_1,2_ unit, but also at the C2 carbon atom, which formed T_1,3_ units. Formation of oligoglycerols only took place to a low extent.

### 3.3. ε-Caprolactone Polymerization Using hPES-OH ***2*** in Grafting from

**Figure 4:**
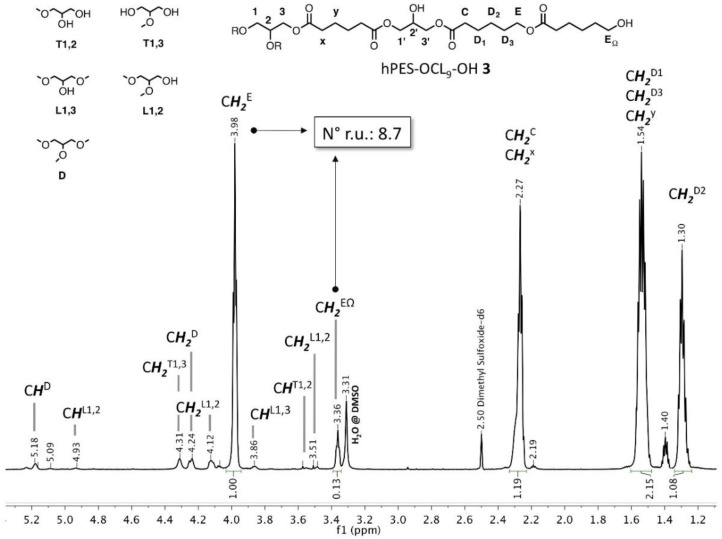
^1^H NMR of hPES-OCL_9_-OH **3** showing peak assignment and crucial methylene signals for determination of number of repeating units of caprolactone.

**Figure A9:**
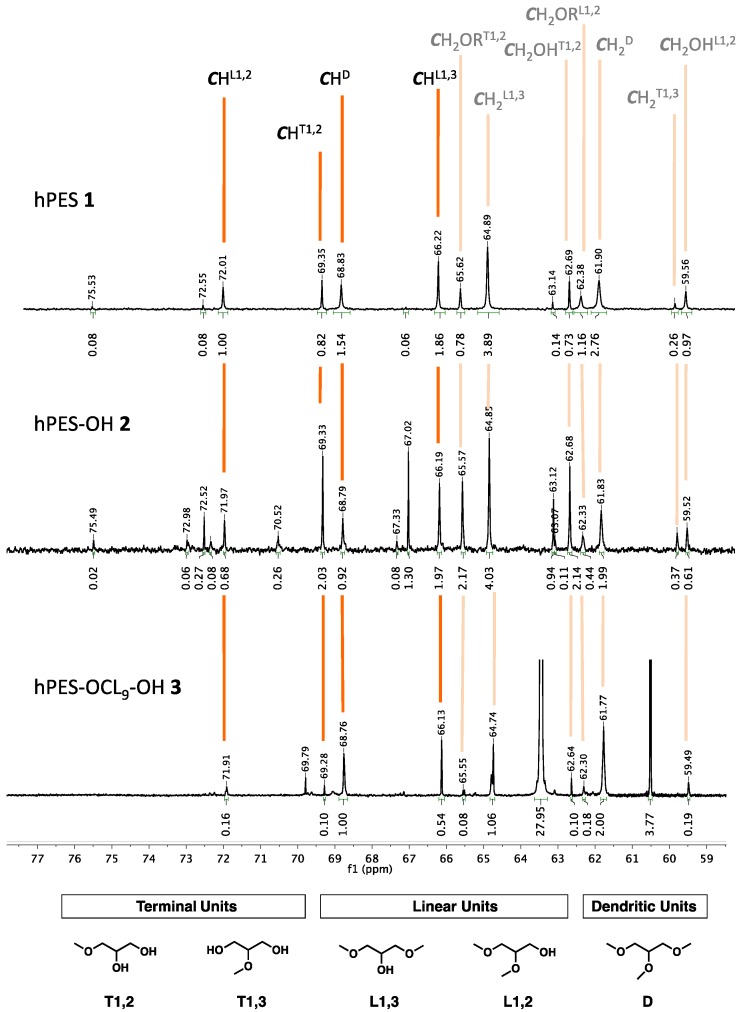
Details of ^13^C NMR spectra of hPES 1, hPES–OH 2, and hPES–OCL_9_–OH 3 (top to bottom). Depicted are peaks relevant for calculation of *D*_f_ and DB analysis. Five types of glycerol units can be distinguished in the structure of the hyperbranched polyester. For comparability of signal integrals, the value for diacid peaks was set to be identical in hPES 1 and hPES–OH 2.

Having hyperbranched polyester hPES-OH **2** at hand, there were two options to synthesize a core-multishell nanocarrier: either by synthesizing the amphiphilic double-shell and attaching it via a grafting*-*to approach, or using the hydroxyl groups of hPES-OH **2** as initiators for a polymerization, aiming at the grafting-from approach. As previous trials with grafting to approaches led to low functionalization of the hyperbranched template, and removal of unreacted amphiphilic double-shell building blocks turned out to be quite tedious, we decided to use the grafting-from approach. As already discussed in other publications, multiple hydroxyl groups on one branched scaffold allow the polymerization to simultaneously take place at ideally all accessible hydroxyl groups [23,24]. This concept of a so-called macroinitiator was used in our herein-presented work to polymerize ε-caprolactone in a tin-catalyzed ring opening polymerization. We aimed for an oligocaprolactone with five repeating units per free OH groups, but as calculated based on ^1^H NMR spectroscopy we, instead, obtained nine repeating units (see Figure 4). The number of repeating units was calculated based on a comparison of methylene CH_2_^E^, at 3.98 ppm and methylene neighboring terminal alcohol groups CH_2_^EΩ^ at 3.36 ppm, which was obtained by ^1^H NMR spectroscopy. In order to investigate whether ring opening occurred at both species of hydroxyl groups of the core’s glycerol units, namely, primary and secondary, we integrated the individual methine CH signals via IG ^13^C NMR and compared their abundance as shown in Table A3. We could show that polymerization occurred at both primary and secondary hydroxyl groups, and the degree of functionalization was *D*_f_ (OH) = 0.67 (for NMR spectra, see Figure A9).

### 3.4. Synthesis of CMS Nanocarrier

**Scheme 3:**
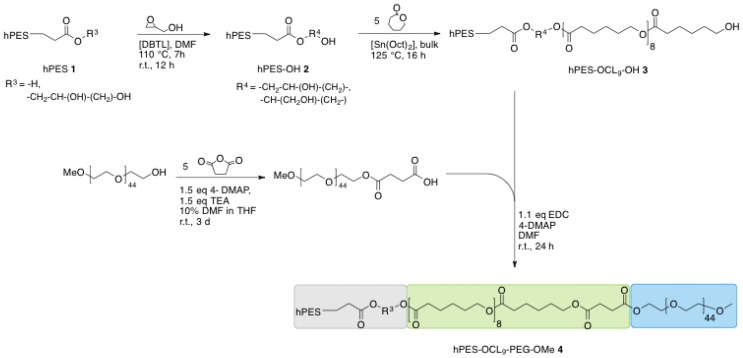
Synthesis of CMS nanocarrier and its building blocks.

The attachment of succinic acid anhydride-modified mPEG outer shell was accomplished by ester bond formation using modified Steglich type conditions, as shown in Scheme 3 [25,26]. We used the water soluble carbodiimide coupling reagent EDC and catalytic amounts of 4-(dimethylamino)-pyridin (4-DMAP) in anhydrous DMF for the ester bond formation, which was followed by a multistep purification approach consisting of extraction and ultrafiltration to remove starting material and low-functionalized CMS nanocarriers. We used ultrafiltration membranes with a molecular weight cutoff of 10 kDa, which yielded a product with a quite narrow molecular weight distribution of *M*_w_/*M*_n_ = 1.1. The decreasing PDI from the precursor hPES-OH **2** and hPES-OCL9-OH **3** to the final product hPES-OCL_9_-mPEG 4 reflects the effort put into the purification procedure. While hPES-OH **2** was not purified, hPES-OCL_9_-OH **3** was purified by precipitation and dialysis, removing products with very high and very low functionalization. The impact of purification on the PDI can be observed especially in the final product hPES-OCL_9_-mPEG **4**, where a combination of ultrafiltration and precipitation was performed. As described in Materials and Methods, the ultrafiltration desks had a molecular weight cutoff of 10 kDa and, hence, allowed the removal of low molecular weight compounds. To further narrow down the molecular weight distribution, fractionation was performed. This strategy consisting of precipitation and dissolution including heat-chill cycles lead to the removal of high molecular weight side-products. The initial PDI of 1.2 obtained after ultrafiltration was therefore decreased to 1.1. In addition to the impact of the purification, it is known and has been reported in the literature that the PDI of CMS nanocarriers is often lower than the PDI of the core or core-single shell precursors [24,27]. Owing to both the multistep synthesis and the extensive purification procedures, the overall yield was 12%. ^1^H NMR measurements of the purified product **4** were analyzed with respect to increasing the ester neighboring methylene signals, which revealed an esterification efficacy of oligocaprolactone’s hydroxyl groups of *D*_f_ (CL) = 0.7. Furthermore, we could show that 55% of present mPEG chains were attached to terminal caprolactone and 45% of mPEG chains reacted with internal glycerol units. The *M*_n_ of the final CMS nanocarrier as calculated by NMR of 30 kDa is similar to the findings from GPC measurement, which were obtained by comparison to linear polymer standards. Table 1 summarizes the characteristics of the CMS nanocarrier and its building blocks. The degree of branching increased from 0.41 (hPES-OCL-OH **3**) to 0.52, which matches well the observation that mPEG-COOH reacted with the internal glycerol hydroxyl groups. The size of the CMS nanocarriers in dH_2_O was determined by DLS measurements and showed a hydrodynamic diameter of 28 nm in the volume distribution. The size indicates aggregation behavior of the CMS nanocarriers, which has been observed before [8,28,29,30].

### 3.5. Encapsulation of Dexamethasone in CMS Nanocarriers

In order to elaborate whether polyester-based CMS nanocarriers are suitable for hydrophobic drugs, synthesized CMS nanocarriers were tested for the encapsulation of dexamethasone. Dexamethasone is an approved glucocorticoid drug used for its anti-inflammatory and immune suppressant effect. Due to its hydrophobic nature, it has a low water solubility of *c* = 0.08 mg·mL^−1^ that limits its application. Therefore, solubilizing dexamethasone in water-soluble nanocarriers is of great interest and might increase its significance as a used drug. The encapsulation of dexamethasone is performed according to the film-method uptake. For example, a dry film of 50 wt % dexamethasone is dissolved in a stock solution of CMS in dH_2_O and the dispersion is stirred for 22 h at 1200 rpm, which is followed by filtration using a syringe filter. The filtration process removes big crystals of dexamethasone, while dexamethasone-loaded CMS as well as the water-soluble fraction of dexamethasone pass the filter. Analysis by HPLC combined with an internal UV absorption detector was used to determine the concentration of dexamethasone. The overall concentration of dexamethasone of the CMS nanocarrier sample is *c* = 0.182 mg·mL^−1^. This value roughly correlates to one molecule of encapsulated dexamethasone per CMS nanocarrier after deducting the drug’s natural solubility.

### 3.6. Acidic and Enzymatic Degradation of CMS Nanocarriers

**Figure 5:**
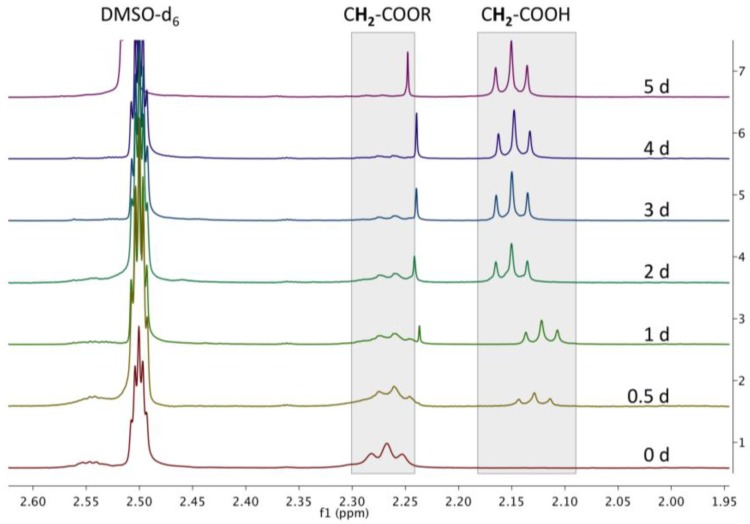
Overlay of magnified ^1^H NMR spectra in DMSO-d_6_ of CMS nanocarriers after incubation with CAL B at 32 °C at various time points during a five-day study; simultaneous decrease of –C**H_2_**–COOR at 2.27 ppm and increase of –C**H_2_**–COOH at 2.15 ppm.

**Figure A10:**
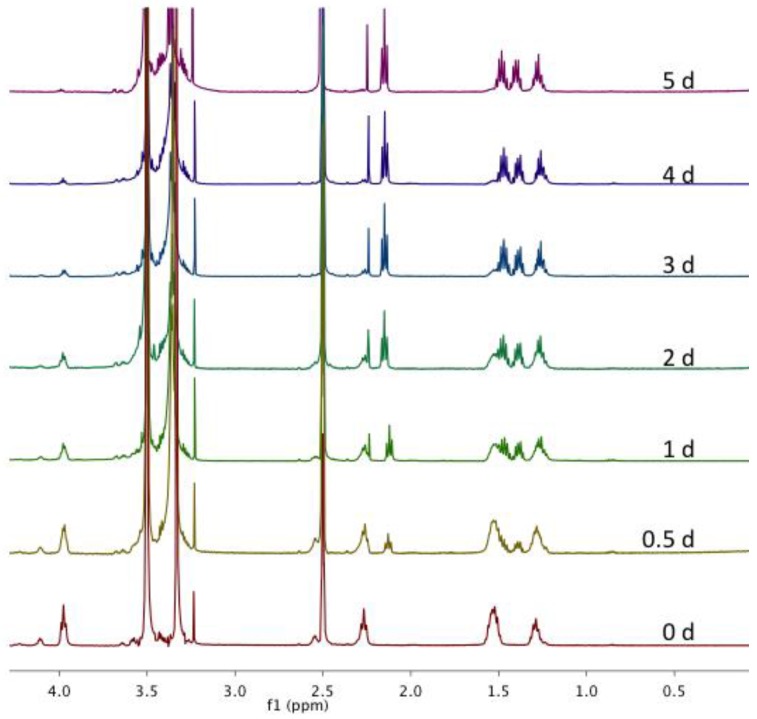
Overlay of magnified ^1^H NMR spectra in DMSO-d_6_ of CMS nanocarriers after incubation with Novozyme^®^ 435 at 32 °C at various time points during a five-day study.

In order to mimic the conditions in skin, degradation of CMS nanocarriers was investigated in both acidic and enzymatic environments at 32 °C. For creating an acidic environment, CMS nanocarriers were solubilized in an acetic acid buffer of pH 5.0, while the enzymatic degradation study was performed using non-specific lipase expressed from Candida Antarctica (CAL B) and immobilized on acrylic resin [31,32]. Both studies were performed for five days and sampled at defined time points. A control experiment at pH 7.4 in the absence of enzyme was performed simultaneously and showed no degradation. The hydrolysis of ester bonds leading to formation of carboxylic acids was monitored by ^1^H NMR spectroscopy in DMSO-d_6_. While the signal intensity of methylene-neighboring ester bonds at 2.3 ppm decreased, the intensity of methylene neighboring carboxylic acids at 2.2 ppm increased, as depicted in Figure 5. After one day of incubation, a new peak at 2.24 ppm appeared, which is the signal of cleaved succinic acid. Even though the integral of this signal does not allow for isolated integration of the peak at 2.3 ppm, the disappearance of the ester-neighboring methylene is clearly visible. Table 2 gives the values of the individual integrals for each time point, as well as the percentage of cleaved ester bonds. After five days of enzymatic degradation, nearly all ester bonds are degraded (for full spectrum, please see Figure A10). In contrast to the successful enzymatic degradation, no degradation was observed in acidic environment at pH 5.0, even after seven days.

## 4. Conclusions

A hyperbranched polyester was synthesized in a bulk polycondensation based on adipic acid and glycerol, catalyzed by the tin catalyst DBTL. The obtained branched scaffold with a degree of branching of 0.41 and a rather low molecular weight of *M*_n_ = 900 Da was modified with glycidol in a ring-opening reaction in the presence of the tin catalyst. The obtained scaffold was further used as a macroinitiator for the ring-opening polymerization of ε-caprolactone. Based on ^13^C NMR spectroscopy, we showed that the polymerization of ε-caprolactone was initiated by both primary and secondary hydroxyl groups of mainly terminal glycerol units, which led to oligocaprolactone chains with about nine repeating units and an overall degree of functionalization of 0.67. After functionalization of the obtained scaffold with mPEG–COOH linear chains via ester bond formation using modified Steglich type conditions followed by purification, we obtained CMS nanocarriers with a *M*_n_ of 28,000 Da and a low dispersity of 1.1. While 60% of all attached mPEG chains were bound to caprolactone chains ends, another 40% were attached to internal glycerol hydroxyl groups. The obtained water-soluble CMS nanocarrier was used for the encapsulation of the hydrophobic drug dexamethasone using the film method uptake and resulted in a transport capacity of one molecule dexamethasone per CMS nanocarrier. Furthermore, we performed degradation studies showing almost full degradation within five days at 32 °C, mediated by lipase, while degradation in acidic environment at pH 5.0 within seven days at 32 °C was not observed.

## Figures and Tables

**Table 1:** Comparison of physical characteristics of CMS with its macroinitiator and intermediate.

Compound	*D*_f_^a^	DB ^a^	*M*_n_^b^	*M*_w_/*M*_n_^b^
hPES-OH **2**	0.83	0.41	900 ^b^	2.7 ^b^
hPES-OCL_9_-OH **3**	0.67	0.74 ^c^	5,150 ^d^	1.7 ^d^
hPES-OCL_9_-mPEG **4**	0.7 ^e^	0.52	27,900 ^d^	1.1 ^d^

^a^ determined via NMR; ^b^ GPC in THF, Pullulan standards; ^c^ based on overnight ^13^C NMR measurements; ^d^ GPC in DMF, PS standards; and ^e^ *D*_f_ of terminal caprolactone hydroxyl groups.

**Table 2:** Degree of degradation of CMS nanocarriers by incubation with CAL B.

Incubation time (Days)	Ratio C*H_2_*–COOR: C*H_2_*–COOH	Degree of degradation (%)
0	1:0	0
0.5	1:0.3	24
1	1:0.4	30
2	1:4.3	81
3	1:5.2	84
4	1:6	86
5	1:22	96

**Figure A1:**
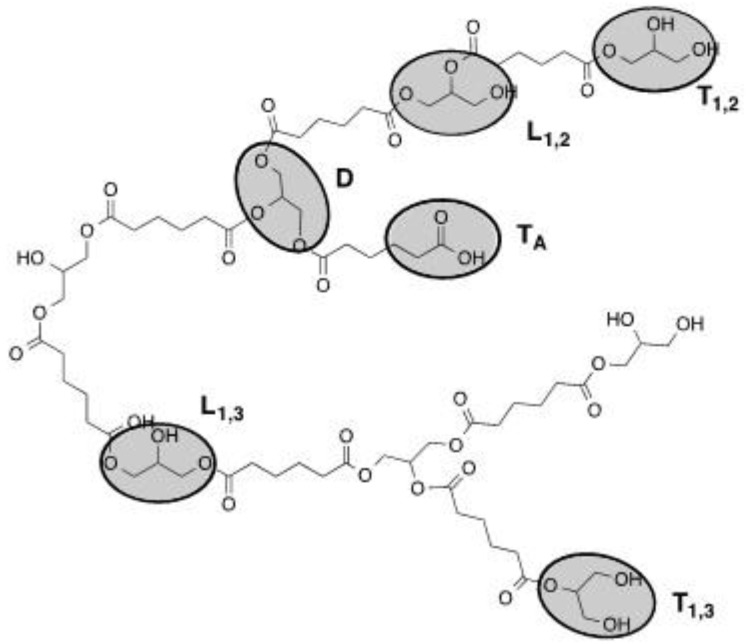
Structural units of hPES **1**.

**Figure A2:**
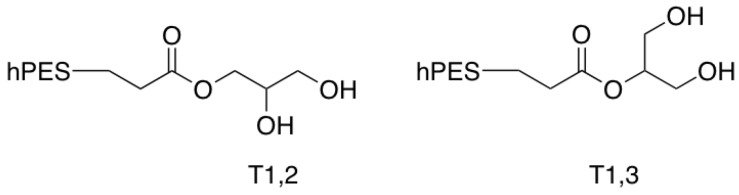
Terminal glycerol units of hPES-OH 2.

**Figure A3:**
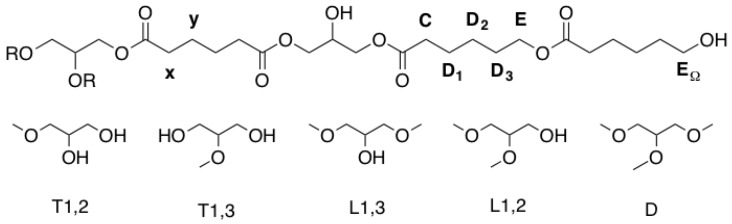
Labelling of atoms and structural units of hPES-OCL_9_-OH 3 used for NMR description.

**Figure A4:**
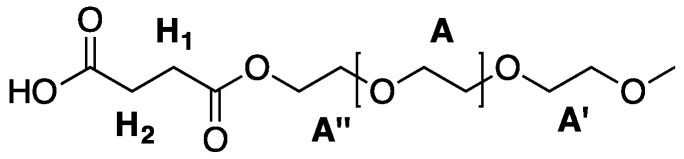
Labelling of atoms of mPEG-COOH used for NMR description.

**Figure A5:**
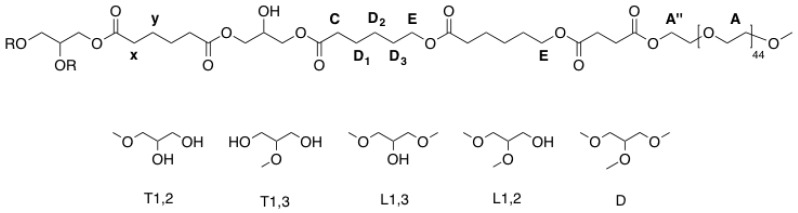
Labelling of atoms and structural units of hPES-OCL_9_-OH 3 used for NMR description.

**Scheme A1:**
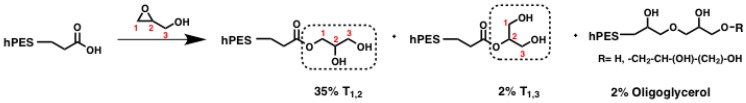
Simplified scheme of polyester modification by ring-opening reaction of glycidol; crucial units for NMR evaluation are highlighted in boxes. %: abundance of species relative to abundance of all glycerol units.

## Abbreviations

The following abbreviations are used in this manuscript:

NMR: nuclear magnetic resonance
PEG: poly(ethylene glycol)
CMS: core-multishell nanocarrier
ε-CL: ε-caprolactone
mPEG: methoxy poly(ethylene glycol)
Sn(Oct)_2_: Tin(II) 2-ethylhexanoate
DBTL: dibutyltin dilaurate
EDC: 1-(3-dimethylaminopropyl)-3-ethylcarbodiimide hydrochloride
4-DMAP: 4-(dimethylamino)-pyridin
MWCO: molecular weight cut-off
TAV: total acid value
THV: total hydroxyl value
GPC: gel permeation chromatography
SEC: size-exclusion chromatography
PMMA: poly(methyl methacrylate)
HPLC: high pressure liquid chromatography
hPES: hyperbranched poly (ester)
eq.: equivalents
IG: inverse-gated
DCM: dichloromethane
MeOH: methanol
TEA: triethylamine
Et_2_O: diethyl ether
DMF: dimethylformamide
dH_2_O: deionized water
ACN: acetonitrile
LC: loading capacity
*P*_A_: conversion of polymerization at gel point
DB: degree of branching
HMQC: Heteronuclear multiple-quantum correlation spectroscopy
ROP: ring opening polymerization
PCL: poly(ε-caprolactone)
r.u.: repeating units
rpm: revolutions per minute
DMSO: dimethyl sulfoxide
*M*_w_: weight average molecular weight
*M*_n_: number average molecular weight

## Appendix

### A.1. Synthesis of Hyperbranched Polyester (hPES ***1***)

At room temperature, adipic acid (39.9 g, 273 mmol, 1.2 eq) was charged into a three-neck glass vertical reactor, equipped with a mechanical stirrer and a Liebig condenser. After adding pre-dried glycerol (20.9 g, 228 mmol), the bulk monomer mixture was heated to 150 °C. Under stirring at 150 rpm, a 0.6 mL of a stock solution of DBTL in toluene (100 ppm) was added to the molten monomers using a syringe. The reaction temperature was increased to 160 °C. After 1 h at 160 °C, the formed volatiles were removed by cryo-distillation and collected in a round-bottom flask. The removal of volatiles was repeated every hour. With proceeding reaction time, the frequency of volatile removal was increased to once per every 10 min. Conversion of the reaction was controlled by determining the ratio of the unreacted acid groups to the total amount of acid groups, using ^1^H NMR spectroscopy. When the conversion almost reached the maximum conversion *P*_A_. as determined by Flory Equation, the reaction was stopped by completely removing the volatiles and cooling the reactor to RT. The viscous product hPES **1** was obtained without any further purification as a light yellow, viscous solid and dissolved in THF for easier handling.

**^1^H NMR** (400 MHz, DMSO-d_6_): δ = 12.00 (s, 1H, R–COO**H**), 5.18 (m, 1H, C**H**^D^), 4.94 (m, 1H, C**H**^L1,2^), 4.72 (m, 1H, C**H**^T1,3^), 4.26–4.23, 4.14–4.12 (2 m, 4H, C**H*_2_***^D^), 4.23–4.07 (m, 2H, C**H*_2_***^L1,2^), 4.03, 3.89 (2 m, 4H, C**H*_2_***^T1,3^), 3.98 (m, 4H, C**H*_2_***^L1,3^), 3.86 (m, 1H, C**H**^L1,3^), 3.63 (q, 1H, C**H**^T1,2^), 3.49–3.40 (m, 4H, C**H*_2_***^T1,3^), 3.49–3.48 (m, 2H, C**H*_2_***^L1,2^), 3.55–3.26 (m, 2H, C**H_2_**^T1,2^), 2.30 (m, 2H, –C**H*_2_***–COOR), 2.20 (m, 2H, –C**H*_2_***–COOH), 1.52 (m, 4H, ROOC–CH_2_–(C**H*_2_***)_2_–CH_2_–COOR) ppm. Abbreviations in accordance with Figure A1.

**IG** **^13^C** **NMR** (400 MHz, DMSO-d_6_): δ = 174.42–174.34 (m, 1H, HOO**C**–(CH_2_)_4_–COO–CH_2_–(CHOR)–(CH_2_OR)), 172.88–172.08 (m, HOO**C**–(CH_2_)_4_–COO–CH_2_–(CHOH)–(CH_2_OR), HOOC–(CH_2_)_4_–**C**OO–CH_2_–(CHOH)–(CH_2_OR), HOOC–(CH_2_)_4_–**C**OO–CH_2_–(CHOR)–(CH_2_OR), (CH_2_OR)–(CHOR)–CH_2_–OO**C**–(CH_2_)_4_–**C**OO–CH_2_–(CHOR)–(CH_2_OR)), 75.53 (s, 1 C, **C**H ^T1,3^), 72.01 (s, 1 C, **C**H^L1,2^), 69.35 (s, 1 C, **C**H^T1,2^), 68.83 (s, 1 C, **C**H^D^), 66.22 (s, 1 C, **C**H^L1,3^), 65.62 (s, 1 C, **C**H_2_–OR^T1,2^), 64.89 (s, 1 C, **C**H_2_^L1,2^), 62.69 (**C**H_2_–OH^T1,2^), 62.38 (s, 1 C, **C**H_2_–OR^L1,2^), 61.90 (s, 1 C, **C**H_2_^D^), 59.85 (s, 1 C, **C**H_2_^T1,3^), 59.56 (s, 1 C, **C**H_2_–OH^L1,2^), 33.44–32.93 (m, 2 C, ROCO–**C**H_2_–(CH_2_)_2_ –**C**H_2_–COOR), 24.10–23.67 (m, 2 C, ROCO–CH_2_–(**C**H_2_)_2_–CH_2_–COOR) ppm. Abbreviations in accordance with Figure A1.

**Table A1:** Molecular weight distribution of hPES **1**.

Peak No.	*M*_n_	*M*_w_	*M*_w_/*M*_n_
1	3.4 × 10^7^ Da	4.2 × 10^7^ Da	1.2
2	300 Da	1,600 Da	5

**DB:** 0.52

**GPC** (THF)

**TAV:** 1.80 mmol COOH/g polymer

**THV:** 2.25 mmol OH/g polymer

**IR**: ν = 3458.71, 2948.63, 1729.83, 1455.03, 1416.46, 1381.75, 1166.72, 1135.87, 1078.01, 1061.62, 943.02, 754.031 cm^−1^.

### A.2. Synthesis of Polyesterol hPES-OH ***2***

30 mL of a solution of hyperbranched polyester **hPES 1** in THF (*c* = 347 mg·mL^−1^, 10.41 g, 19 mmol COOH) was charged into a Schlenk flask. The residual catalyst DBTL (1.3 mg, 3.9 μmol) from the original product **hPES** **1** was used to catalyze the reaction; no further catalyst was added. After solubilization in 10 mL DMF under stirring at RT, the flask was heated to 85 °C. THF was removed from the mixture under controlled reduced pressure using cryo-distillation, and the completeness of the THF removal was controlled by ^1^H NMR. After THF was removed, the mixture was heated to 110 °C. Glycidol (1.25 mL, 1.388 g, 19 mmol, 1 eq) was added drop wise to the stirring yellow solution during a time period of 10 min using a syringe. The reaction mixture was stirred at 110 °C for 120 min, afterwards at RT overnight. Due to the incompleteness of reaction, the reaction was reheated to 110 °C and more glycidol (0.1 mL, 20 mmol in total) was added dropwise to the stirring reaction mixture. The reaction was stirred at 110 °C for 5.5 h and afterwards allowed to cool down to RT. The viscous product was obtained without further purification and stored in DMF.

**^1^****H** **NMR** (500 MHz, DMSO-d_6_): δ = 7.95 (s, DMF), 5.18 (s, 1 H, C***H***^D^), 4.94 (s, 1 H, C***H***^L1,2^), 4.72 (q, 1 H, C***H***^T1,3^), 3.86–4.26 (m, 13 H, C***H***^L1,3^, C***H****_2_*^T1,2^, C***H****_2_*^L1,3^, C***H****_2_*^L1,2^, C***H****_2_*^D^), 3.63 (q, 1 H, C***H***^T1,2^), 3.60 (THF), 3.27–3.50 (m, 6 H, C***H****_2_*^’T1,2^, C***H****_2_*^T1,3^, C***H****_2_*^’L1,2^), 3.19, 3.02, 2.89, 2.73 (s, DMF), 2.59, 2.31 (m, 2H, –C***H****_2_*–CO_2_–R), 2.10–2.12 (m, 2 H, –C***H****_2_*–CO_2_H), 1.75 (THF), 1.54 (m, 4 H, –OCO–CH_2_–(C***H****_2_*)_2_–CH_2_–COO–) ppm. Abbreviations in accordance with Figure A1 and Figure A2.

**IG** **^13^C** **NMR** (500 MHz, DMSO-d_6_): δ = 174.93–175.04 (CH_2_–***C***OOH), 172.08–172.85 (CH_2_–***C***OOR), 75.49 (***C***H^T1,3^), 72.98, 72.52, 71.97 (***C***H^L1,2^), 70.52, 69.33 (***C***H^T1,2^), 68.79 (***C***H^D^), 67.02 (THF), 66.19 (***C***H^L1,3^), 65.57 (***C***H_2_–OR^T1,2^), 64.85 (s, ***C***H_2_^L1,3^), 62.68 (s, ***C***H_2_–OH^T1,2^), 62.33 (s, ***C***H_2_–OR^L1,2^), 61.83 (s, ***C***H_2_^D^), 59.79 (***C***H_2_^T1,3^), 59.52 (***C***H_2_–OH^L1,2^), 35.75 (DMF), 32.85–33.36 (–OCO–***C***H_2_–(CH_2_)_2_–***C***H_2_–COO), 30.74 (DMF), 25.13 (THF), 23.62–23.91 (–OCO–CH_2_–(***C***H_2_)_2_–CH_2_–COO–) ppm. Abbreviations in accordance with Figure A1 and Figure A2.

**DB**: 0.41

**GPC:** *M*_n_ = 900 Da; *M*_w_ = 2400 Da, *M*_w_/*M*_n_ = 2.7

**TAV:** 0.045 mmol COOH/g polymer

**THV:** 5.3 mmol OH/g polymer

**IR:** ν = 3384.46, 2938.02, 2871.49, 2332.48, 1732.73, 1660.41, 1439.6, 1409.71, 1383.68, 1250.61, 1167.69, 1137.8, 1091.51, 1054.87, 937.24, 865.88 cm^−1^.

### A.3. Synthesis of Linear Di-Block Copolymer hPES-OCL_9_-OH ***3***

In a Schlenk flask pre-dried macroinitiator hPES–OH **2** (2 g, 10.6 mmol OH) was dissolved in freshly distilled ε-caprolactone (6.18 g, 54 mmol) at 60 °C and two drops of Sn(Oct)_2_ were added to the stirring mixture, followed by an increase of the temperature to 125 °C. The bulk mixture was stirred at 125 °C for 18 h. Purification was performed by dissolving the crude reaction mixture in DCM in high dilution and precipitation in a high excess of ice-cold MeOH under vigorous stirring. The dispersion was separated from the formed gel, solvent was removed under reduced pressure, and the received solid was redissolved in DCM in high dilution. Another precipitation was performed by adding the DCM solution dropwise into vigorously stirred ice-cold diethyl ether. The mixture was separated by centrifugation at 4000 min^−1^ for 1 min, and the supernatant was collected. After drying the separated supernatant under reduced pressure, the received solid was once again purified using dialysis in DCM (benzoylated RC membrane, MWCO 1–2 kDa, 7 h) for removal of Sn(Oct)_2_ and traces of ε-caprolactone. A white, wax-like solid product was obtained after removing the solvent under reduced pressure (2.69 g, yield: 33%).

**^1^H** **NMR** (500 MHz, DMSO-d_6_): δ = 5.18 (C***H***^D^), 5.09 (new), 4.93 (C***H***^L1,2^), 4.31 (C***H_2_***^T1,3^), 4.24 (C***H_2_***^D^), 4.12 (C***H_2_***^L1,2^), 3.98 (C***H_2_***^E^), 3.86 (C***H***^L1,3^), 3.57 (C***H***^T1,2^), 3.51 (C***H_2_***^L1,2^), 3.36 (C***H_2_****^E^*^Ω^), 2.27 (m, C***H_2_***^C^, C***H_2_***^X^), 1.54 (C***H_2_***^D1^, C***H_2_***^D3^, C***H_2_***^y^), 1.40, 1.30 (m, C***H_2_***^D2^) ppm. Abbreviations in accordance with Figure A3.

**^13^C** **NMR** (700 MHz, overnight, DMSO-d_6_): δ = 172.8, 172.7, 172.4 (various CH_2_-***C***OOR), 71.9 (***C***H^L1,2^), 69.8 (new), 69.3 (***C***H^T1,2^), 68.8 (***C***H^D^), 66.1 (***C***H^L1,3^), 64.7 (***C***H_2_^L1,3^), 63.5 (***C***H_2_^E^), 62.6 (***C***H_2_OH^T1,2^), 62.3 (***C***H_2_OR^L1,2^), 61.8 (***C***H_2_^D^), 60.5 (***C***H_2_^EΩ^), 59.5 (***C***H_2_OH^L1,2^), 33.6 (***C***H_2_^X^), 33.3 (***C***H_2_^C^), 32.1, 27.8 (***C***H_2_^D3^), 25.0, 24.9 (***C***H_2_^D2^), 24.4, 24.1 (***C***H_2_^D1^) ppm. Abbreviations in accordance with Figure A3.

**DB:** 0.74

**GPC:** *M*_n_ = 5000 Da, *M*_w_ = 8900 Da, *M*_w_/*M*_n_ = 1.72

### A.4. Functionalization of mPEG-OH (mPEG-COOH)

To a solution of pre-dried mPEG (4.786 g, 2.5 mmol) in a mixture of 10% anhydrous DMF in anhydrous THF (28 mL) in a 50 mL Schlenk flask, 4-DMAP (0.44 g, 3.8 mmol, 1.5 eq), TEA (0.5 mL, 3.8 mmol, 1.5 eq), and succinic anhydride (1.2 g, 12.5 mmol, 5 eq) were added under stirring at RT After stirring at RT for three days, the unreacted precipitated starting material was removed from the solution and the solution was dried under reduced pressure. The crude product was purified by precipitation from DCM solution into an ice-cold, 10-fold excess of Et_2_O. The formed precipitate was filtered off using a glass filter (P4), redissolved in DCM, and precipitated once more following the same procedure. The collected precipitate was dried under high vacuum and 3.5 g (1.67 mmol, yield: 70%) of pure product were obtained.

**^1^H** **NMR** (500 MHz, DMSO-d_6_): δ = 4.21 (t, 2 H, C***H_2_***^A″^), 3.73–3.45 (m, 185 H, various C***H_2_***^A^), 3.33 (s, 3 H, PEG–O–C***H_3_***), 2.64–2.58 (m, 4 H, C***H_2_***^H1^, C***H_2_***^H2^) ppm. Abbreviations in accordance with Figure A4.

### A.5. Synthesis of Core-Multishell Nanocarrier (hPES-OCL_9_-PEG-OMe ***4***)

In a dried 25 mL Schlenk flask, solid mPEG–COOH (1.110 g, 0.529 mmol, 1.1 eq) was added at RT to a stirring solution of hPES–OCL_9_–OH **3** (304 mg, 0.48 mmol OH) in 6 mL anhydrous DMF. After the addition of 4-DMAP (0.016 g, 0.106 mmol, 20 mol %), EDCl (0.110 g, 0.574 mmol, 1.1 eq) was added at 0 °C. The reaction mixture was stirred for 10 min at 0 °C and then allowed to reach RT by removing the ice bath. After 20 h of stirring at RT, the crude product was purified via extensive ultrafiltration (DMF, MWCO < 10 kDa), followed by repeated fractionation. For this purpose, the impure product was dissolved in DCM, which yielded a clear solution. Hexane was added to the clear solution at RT until cloudiness appeared. The cloudy dispersion was heated to obtain a clear solution, followed by the addition of Hexane to obtain a dispersion. The warm solution was allowed to reach RT and centrifuged (1 min, 3900 min^−1^) to separate into a stable dispersion and sediment. The dispersion was dried and refractionated following the above-described procedure. The progress of purification was monitored using GPC in DMF. After six cycles of refractionation and removal of solvent under reduced pressure, followed by drying at high vacuum, a white solid product was obtained (0.116 g, yield: 12%).

**^1^H NMR** (700 MHz, DMSO-d_6_): δ = 5.17 (C***H***^D^), 4.24 (C*H_2_*^D^), 4.12 (C***H_2_***^L1,2^), 3.98 (C***H_2_***^E^), 3.59–3.40 (various C***H_2_***^A^, C***H_2_***^A″^), 3.24 (PEG–OC***H_3_***), 2.27 (C***H_2_***^C^, C***H_2_***^x^), 1.54 (C***H_2_***^D1^,C***H_2_***^D3^, C***H_2_***^y^), 1.29 (C***H_2_***^D2^) ppm. Abbreviations in accordance with Figure A5.

**^13^C NMR** (500 MHz, DMSO-d_6_): δ = 172.7 (–***C***OOR–), 171.9 (–***C***OOR–), 78.9, 75.3, 71.3 (***C***H^L1,2^), 69.8 (PEG backbone), 69.6, 68.22 (various ***C***H_2_, ***C***H_2_), 69.6 (***C***H^D^), 63.8 (***C***H_2_^L1,2^), 63.5 (***C***H_2_^E^), 61.8 (***C***H_2_^D^), 58.0 (PEG–O***C***H_3_), 33.3–33.1 (***C***H_2_^C^, ***C***H_2_^x^), 28.5 (***C***H_2_^H1^, ***C***H_2_^H2^), 27.8 (***C***H_2_^D1^, ***C***H_2_^D3^), 24.9 (***C***H_2_^D2^), 24.1 (***C***H_2_^y^) ppm. Abbreviations in accordance with Figure A5.

**DB:** 0.52

**GPC:** *M*_n_ = 27,900 Da, *M*_w_ = 31,100 Da, *M*_w_/*M*_n_ = 1.11

### A.7. Determination of TAV and THV of hPES **1** and hPES-OH **2**

To determine the total acid value (TAV) and total hydroxyl value (THV) it is crucial to know the conversion at the respective point of the reaction. Synthesis of hyperbranched polyester **1** consisting of a diacid (A2) and triol (B3) was stopped before reaching the so-called gel point. According to Flory, the gel point is the point when the conversion reaches its maximum before becoming an infinite network [14]. The actual gel point can be predicted as a function of the functionality *f* and ratio ρ. In our case of adipic acid as A_2_ and glycerol as B_3_ unit, functionality *f* is 3, reflecting three functional groups on the branching unit, while ratio ***ρ*** is the ratio between the number of A functional groups and the number of B functional groups yields:

(A1) $$\rho =\frac{n_{\text{COOH}}}{n_{\text{OH}}}$$

*n_CO_*_OH,_ amount of acid groups in mol; *n*_OH,_ amount of hydroxyl groups in mol.

This theoretical approach does not take into account the difference in reactivity of primary and secondary functional groups of the B_3_ trifunctional branching unit. Functionality *f* can be used to determine the critical value α***_c_*** of the branching coefficient α for formation of infinite networks:

(A2) $$\alpha_{c}=\frac{1}{f-1}$$

*f*, functionality of branching unit; α_c_, critical value of branching coefficient α

In our case for B_3_ monomers, *f* is 3 and α_c_ consequently 0.5. The connection between functionality *f* and ratio ρ is as follows:

(A3) PA=(αcρ)12

*P*_A_, conversion at gel point; α_c_, critical value of branching coefficient α; ρ, ratio, see above.

Equation (3) allows for prediction of the theoretical conversion P_A_ at the gel point. For the synthesis of hPES **1** with a ratio of 1.2:1 (A_2_:B_3_), the value for P_A_ is 0.79. The conversion during the reaction was monitored by using ^1^H NMR spectroscopy and analysis of the ratio of C**H_2_**–COOH (2.3 ppm) versus C**H_2_**–COOR (2.2 ppm). When the reaction approached the theoretical P_A_ value, the reaction was stopped and the bulk polymer cooled down to stop the polymerization. After evaluation of the real P value, the TAV and THV were calculated for hPES **1** as follows:

*m*(adipic acid) = 39.9 g *n*_0_(COOH) = 545 mmol
*m*(glycerol) = 20.93 g *n*_0_(OH) = 682 mmol
*m*(polymer) = 52.708 g

Conversion *p* = (Integral C**H_2_**–COOH)/(Integral C**H_2_**–COOH + Integral C**H_2_**–COOR) = 1/1.21 = 0.83

TAV:

*n_t_*(COOH) = (1−P) × *n*_0_(COOH) = 94.9 mmol

➔TAV = 94.9 mmol COOH/52.708 g polymer = 1.80 mmol/g polymer

THV:

*n_t_*(OH) = (1−P) × *n*_0_(OH)= 118.7 mmol

➔THV = 118.7 mmol OH/52.708 g polymer = 2.25 mmol/g polymer

Values of TAV and THV of hPES–OH **2** were determined based on the assumption that COOH reacted with glycidol with a conversion of *p* = 0.97.so that hPES–OH **2** had 2.9% remaining COOH:

*n*_0_(COOH) = 18.7 mmol

*n*_0_(OH) = 26.8 mmol

*m*(polymer) = 11.918 g

TAV:

*n_t_*(COOH) = (1−P) × *n*_0_(COOH) = 0.5423 mmol

➔TAV = 0.5423 mmol/11.918 g polymer = 0.045 mmol COOH/g polymer

THV:

*n_t_*(OH) = *n*_0_(OH) + [(*n*_0_(COOH) − *n_t_*(COOH)) × 2] = 63.1 mmol

➔THV = 63.1 mmol OH/11.918 g polymer = 5.3 mmol OH/g polymer

### A.8. Determination of Degree of Branching DB

The degree of branching was calculated based on Equation (4), which is the calculation of the DB as published by Frey *et al.* We chose this equation, because Frey states that the original Fréchet Equation (5) overestimates the branching, if small or low branched molecules are considered [12,33].

(A4) $$DB=\frac{2\;D}{2\;D+L}$$

(A5) $$\;\;DB=\;\frac{D+T}{L+D+T}$$

Where: *T*: relative integral of terminal units of type **T_1,2_**, **T_1,3_**, and **T_A._**

*L*: relative integral of linear units of type **L_1,3_** and **L_1,2_**

*D*: relative integral of dendritic unit **D**.

The relative integrals needed for this calculation are the integrals of methine signals of the various glycerol branching units, as depicted in Figure A9 for the case of hPES 1, hPES-OH 2, and hPES–OCL_9_–OH 3. The spectra were measured by inverse-gated ^13^C spectroscopy, because the obtained carbon peaks were quantifiable. Table A2 summarizes all the relevant data for the calculation of branched products 1, 2, 3, and 4. In the case of product CMS 4, a quantification of methine and methylene signals was not possible.

**Table A2:** Interpretation of inverse-gated (IG) ^13^C NMR spectra of hyperbranched polyesters in DMSO-d_6_. Integral values of –CH– signals of relevant glycerol units for evaluating the degree of branching (DB).

Structure	Integral value			DB
	CH ^L1,2^	CH ^D^	CH ^L1,3^	
hPES **1**	1.00	1.54	1.86	0.52
hPES-OH **2**	0.74	1.00	2.15	0.41
hPES–OCL_9_–OH **3**	0.16 *	1.00 *	0.54 *	0.74 *

* obtained from overnight ^13^C NMR measurement, non IG; neither CH ^L1,2^ and CH ^L1,3^ nor CH2 ^L1,2^ and CH2 ^L1,3^ were detected in IG due to the low signal-to-noise ratio.

### A.9. Determination of Degree of Functionalization Df

#### A.9.1. hPES-OH **2**

Hyperbranched polyester hPES **1** with a total acid value of 1.8 mmol carboxylic acid groups per gram polyester was modified with equimolar amounts of glycidol with respect to the amount of hydroxyl groups of hPES **1**, in order to modify one glycidol per carboxylic acid. The reactions were performed at a bath temperature of 110 °C in DMF for two hours; afterwards at room temperature overnight. The reaction progress was monitored by ^1^H NMR, *i.e.*, the disappearance of the methylene signal next to the free carboxylic acid as well as the proton signal of terminating carboxylic acid groups. We paid special attention to the expected changes of several structural units as well as the possibility of the side reaction shown in Scheme A1.

Even though equimolar amounts of glycidol were used during the reaction, full conversion of all carboxylic acid groups could not be achieved. Evaluation of the methylene signals next to free carboxylic acids versus those next to ester groups after ^1^H NMR measurements showed that the fraction of carbons from free carboxylic acid decreased from 17.4% before to 2.9% after modification, giving a functionalization degree of D_f_ (COOH) = 0.83. At the same time, the amount of esterification increased by a factor of 2.5, which was evaluated according to the increased methine peak supplementary inverse-gated ^13^C NMR spectroscopy. Based on inverse-gated ^13^C NMR spectroscopy, we can also show that T^1,2^ methine and methylene signals increased by a factor of 2.5 as well, indicating that these newly formed T^1,2^ units arose from reactions with carboxylic acids at the sterically less-hindered C1 atom of glycidol. We can furthermore observe the increase of T^1,3^ methylene signals by a factor of 1.4. Hence, ring-opening of glycidol also occurred on the sterically more-hindered C2 carbon atom, as shown in Scheme A1. The inverse-gated ^13^C NMR spectra were also checked for further changes of integrals, as we were also interested in the question whether formation of oligoglycerol formation occurred. If terminal glycerol hydroxyl groups initialized ring-opening of glycidol, new signals should have arisen. In fact, three new signals can be found in the region of methine shifts (70.52–72.98 ppm, see Figure A1). With regard to the shift of these signals, they could have resulted from the methylene next to the ether bond between two glycerol units. With an average integral of 0.4, the new signals represent only 2% of the amount of overall carbon signals in the region of glycerol’s methine and methylene signals. As this amount is small, the presence of oligoglycerols should not interfere with further reactions and analysis. In summary, terminal carboxylic acid groups of the hyperbranched polyester hPES **1** were modified with glycerol units by in a tin-catalyzed, ring-opening reaction with glycidol. The amount of esterified carboxylic acid units increased by a factor of 2.5 compared to the esterified carboxylic acid units before the modification, while full modification of carboxylic acids could not be achieved. Ring-opening of glycidol did not only take place at C1 carbon atom leading to a new T^1,2^ unit but also at the C2 carbon atom, which formed T^1,3^ units. Oligoglycerols only formed to a certain extent.

#### A.9.2. hPES-OCL_9_-OH **3**

Determination of *D*_f_ was accomplished based on Equation (6), where the estimated amount of reacted hydroxyl groups of hPES–OCL_9_–OH **3** is compared to the estimated amount of theoretically-available hydroxyl groups of hPES–OH **2**.

(A6) $$D_{f}=\frac{\text{amount reacted}\;\text{OH}\;\text{of hPES}-{\text{OCL}}_{9}-\text{OH }3}{\text{amount free OH of hPES}-\text{OH }2}$$

The amounts of hydroxyl groups were estimated based on the integrals in ^13^C NMR spectra of methine arising from glycerol units, marked with orange lines in Figure A9. The relative abundance of the methine signals was calculated and normalized according to Table A2, which gave the amounts of reacted and free OH grous of hPES–OH 2 and hPES–OCL9–-OH 3 that were needed for Equation (A6):

$$D_{f}=\frac{119-39}{119}=0.67$$

**Table A3:** Comparison of ^13^C NMR spectra of hPES–OH 2 and hPES–OCL_9_–OH 3 as shown in Figure A9 with regard to specific glycerol’s methine signals and calculation of their relative abundance, and absolute and normalized amounts of OH and OR groups for calculation of the degree of functionalization *D*_f_.

	hPES–OH 2					hPES–OCL_9_–OH 3				
**Signal**	CH ^L1,2^	CH ^T1,2^	CH^D^	CH^L1,3^	all	CH ^L1,2^	CH ^T1,2^	CH^D^	CH^L1,3^	all
**abs. integral value**	0.74	2.21	1	2.15	6.1	0.16	0.10	1	0.54	1.80
**rel. integral value**	12%	36%	16%	35%	100%	9%	6%	56%	30%	100%
**amount OH ^a^**	12	72	-	35	119	6.9 ^b^	9.2 ^b^	-	23.1 ^b^	39.3 ^b^
**amount OR**	-	-	48	-	48	-	-	127 ^b^	-	127 ^b^

^a^ Linear units contributed 1 OH group, terminal units contributed two OH groups, dendritic units contributed three OR groups; ^b^ The amount of OH and OR groups was normalized by factor *F* = 0.77 = (119 + 48)/(39 + 127) to maintain a constant sum of OR and OH groups.

#### A.9.3. CMS Nanocarrier hPES–OCL_9_–PEG–OMe **4**

Functionalization of terminal caprolactone hydroxyl groups was analyzed via ^1^H NMR by determining the increase of esterification of caprolactone hydroxyl groups. This was done by comparing the ratio of CH_2_^E^ and CH_2_^C+x^ before the reaction to the ratio after the reaction. Since the integral of CH_2_^C+x^ remained constant upon functionalization with mPEG–COOH, we could fix this integral value and determine the percental increase of CH_2_^E^, leading to a value of D_f_(CL–OH) = 0.7. To cross check this assumption, the integral of methoxy CH_3_ of mPEG was compared to CH_2_^C+x^. This comparison revealed an 80% excess of mPEG chains, which therefore should be attached to internal glycerol hydroxyl groups. The functionalization of internal hydroxyl groups was supported by ^13^C-NMR of the product, which showed a disappearance of CH^L1,2^ and CH^L1,3^ and indicated that the respective hydroxyl groups reacted with mPEG–COOH. As the abundance of internal glycerol units was too small to allow for quantification, we could not estimate the degree of functionalization of internal glycerol hydroxyl groups based on ^13^C NMR. Nevertheless, ^1^H NMR analysis led to the assumption, that roughly 55% of present mPEG–COOH chains were attached to 70% of terminal caprolactone units, while 45% of mPEG–COOH reacted with internal glycerol units.

## References

[1] Miyata K., Christie R.J., Kataoka K. (2011). Polymeric micelles for nano-scale drug delivery. React. Funct. Polym..

[2] Kataoka K., Kwon G.S., Yokoyama M., Okano T., Sakurai Y. (1993). Block copolymer micelles as vehicles for drug delivery (L & J). J. Control. Release.

[3] Kataoka K., Harada A., Nagasaki Y. (2001). Block copolymer micelles for drug delivery: Design, characterization and biological significance. Adv. Drug Deliv. Rev..

[4] Newkome G.R., Moorefield C.N., Baker G.R., Saunders M.J., Grossman S.H. (1991). Unimolecular micelles. Angew. Chem. Int. Ed. Engl..

[5] Kurniasih I.N., Keilitz J., Haag R. (2015). Dendritic nanocarriers based on hyperbranched polymers. Chem. Soc. Rev..

[6] Lukowiak M.C., Thota B.N.S., Haag R. (2015). Dendritic core–shell systems as soft drug delivery nanocarriers. Biotechnol. Adv..

[7] Kadajji V.G., Betageri G.V. (2011). Water soluble polymers for pharmaceutical applications. Polymers.

[8] Fleige E., Ziem B., Grabolle M., Haag R., Resch-Genger U. (2012). Aggregation phenomena of host and guest upon the loading of dendritic core-multishell nanoparticles with solvatochromic dyes. Macromolecules.

[9] Wang X., Gurski L.A., Zhong S., Xu X., Pochan D.J., Farach-Carson M.C., Jia X. (2011). Amphiphilic block co-polyesters bearing pendant cyclic ketal groups as nanocarriers for controlled release of camptothecin. J. Biomater. Sci. Polym. Ed..

[10] Krishnan V., Xu X., Barwe S.P., Yang X., Czymmek K., Waldman S., Mason R.W., Jia X., Rajasekaran A.K. (2013). Dexamethasone-loaded block copolymer nanoparticles induce leukemia cell death and enhance therapeutic efficacy: A novel application in pediatric nanomedicine. Mol. Pharm..

[11] Fleige E., Quadir M.A., Haag R. (2012). Stimuli-responsive polymeric nanocarriers for the controlled transport of active compounds: Concepts and applications. Adv. Drug Deliv. Rev..

[12] Frey H. (1997). Degree of branching in hyperbranched polymers. Acta Polym..

[13] Stumbé J.-F., Bruchmann B. (2004). Hyperbranched polyesters based on adipic acid and glycerol. Macromol. Rapid Commun..

[14] Flory J. (1941). Molecular size distribution in three dimensional polymers. I. Gelation. J. Am. Chem. Soc..

[15] Sunder A., Hanselmann R., Frey H., Mülhaupt R. (1999). Controlled synthesis of hyperbranched polyglycerols by ring-opening multibranching polymerization. Macromolecules.

[16] Luman N.R., Kim T., Grinstaff M.W. (2004). Dendritic polymers composed of glycerol and succinic acid: Synthetic methodologies and medical applications. Pure Appl. Chem..

[17] Zhang T., Howell B.A., Dumitrascu A., Martin S.J., Smith P.B. (2014). Synthesis and characterization of glycerol-adipic acid hyperbranched polyesters. Polymer.

[18] Posner G.H., Rogers D.Z. (1977). Organic reactions at alumina surfaces. Mild and selective opening of epoxides by alcohols, thiols, benzeneselenol, amines, and acetic acid. J. Am. Chem. Soc..

[19] Iranpoor N., Shekarriz M., Shiriny F. (1998). Highly efficient, regio—and stereoselective ring opening of epoxides and thiiranes with Ce(OTf)_4_. Synth. Commun..

[20] Iranpoor N., Tarrian T., Movahedi Z. (1996). FeCl_3_·6H_2_O supported on SiO_2_ catalysed ring opening of epoxides with alcohols, acetic acid, water, chloride, bromide and nitrate ions. Synthesis.

[21] Khalafi-Nezhad A., Soltani Rad M.N., Khoshnood A. (2003). An efficient method for the chemoselective preparation of benzoylated 1,2-diols from epoxides. Synthesis.

[22] Weiss M.E.R., Paulus F., Steinhilber D., Nikitin A.N., Haag R., Schütte C. (2012). Estimating kinetic parameters for the spontaneous polymerization of glycidol at elevated temperatures. Macromol. Theory Simul..

[23] Zhou Y., Huang W., Liu J., Zhu X., Yan D. (2010). Self-assembly of hyperbranched polymers and its biomedical applications. Adv. Mater..

[24] Gao H. (2012). Development of star polymers as unimolecular containers for nanomaterials. Macromol. Rapid Commun..

[25] Steglich W., Höfle G. (1969). *N*,*N*-dimethyl-4-pyridinamine, a very effective acylation catalyst. Angew. Chem. (Int. Ed. Engl.).

[26] Steglich W., Neises B. (1978). Simple method for the esterification of carboxylic acids. Angew. Chem. Int. Ed. Engl..

[27] Fischer A.M., Thiermann R., Maskos M., Frey H. (2013). One-pot synthesis of poly(l-lactide) multi-arm star copolymers based on a polyester polyol macroinitiator. Polymer.

[28] Radowski M.R., Shukla A., von Berlepsch H., Böttcher C., Pickaert G., Rehage H., Haag R. (2007). Supramolecular aggregates of dendritic multishell architectures as universal nanocarriers. Angew. Chem..

[29] Fleige E., Tyagi R., Haag R. (2013). Dendronized core-multishell nanocarriers for solubilization of guest molecules. Nanocarriers.

[30] Fleige E., Achazi K., Schaletzki K., Triemer T., Haag R. (2014). Ph-responsive dendritic core-multishell nanocarriers. J. Control. Release.

[31] Kurniasih I.N., Liang H., Kumar S., Mohr A., Sharma S.K., Rabe J.P., Haag R. (2013). A bifunctional nanocarrier based on amphiphilic hyperbranched polyglycerol derivatives. J. Mate. Chem. B.

[32] Stefani S., Kurniasih I.N., Sharma S.K., Böttcher C., Servin P., Haag R. (2016). Triglycerol-based hyperbranched polyesters with an amphiphilic branched shell as novel biodegradable drug delivery systems. Polym. Chem..

[33] Fréchet J.M.J., Hawker C.J. (1995). Hyperbranched polyphenylene and hyperbranched polyesters: New soluble, three-dimensional, reactive polymers. React. Func. Polym..

